# S-Nitrosylation of p53 in Melanoma Cells Under Nitrosative Stress

**DOI:** 10.3390/ijms26136512

**Published:** 2025-07-06

**Authors:** Mariana Grigoruta, Li Li, Leyuan Chen, Fancui Meng, Kevin P. Rosenblatt, Yiliang Li, Elizabeth A. Grimm, Yong Qin

**Affiliations:** 1 Department of Pharmaceutical Sciences, School of Pharmacy, The University of Texas at El Paso, El Paso, TX 79902, USA; mariana.grigoruta@uacj.mx; 2 Department of Health Sciences, Biomedical Sciences Institute, Autonomous University of Ciudad Juarez, Ciudad Juárez 32310, Mexico; 3 Clinical and Translational Proteomics Service Center, The University of Texas Health Science Center at Houston, Houston, TX 77030, USA; li.li@uth.tmc.edu (L.L.); kevin.rosenblatt@congenx.com (K.P.R.); 4 Tianjin Key Laboratory of Radiation Medicine and Molecular Nuclear Medicine, Institute of Radiation Medicine, Peking Union Medical College & Chinese Academy of Medical Sciences, Tianjin 300192, China; cly1207954768@163.com (L.C.); liyiliang@irm-cams.ac.cn (Y.L.); 5 Tianjin Key Laboratory of Molecular Design and Drug Discovery, Tianjin Institute of Pharmaceutical Research, Tianjin 300301, China; mengfc@tipr.com; 6 Division of Oncology, Department of Internal Medicine, The University of Texas Health Science Center at Houston McGovern Medical School, Houston, TX 77030, USA; 7 Consultative Genomics, PLLC, Bellaire, TX 77401, USA; 8 Department of Melanoma Medical Oncology, MD Anderson Cancer Center the University of Texas, Houston, TX 77030, USA; egrimmroth@gmail.com

**Keywords:** melanoma, nitric oxide, S-nitrosylation, p53, nitrosative stress

## Abstract

Nitric oxide (NO) modifies protein structure and plays a critical role in promoting tumor growth, metastasis, and resistance to therapy. In inflammatory tumors, multiple key proteins, including p53, are susceptible to S-nitrosylation (SNO) by NO. In this study, we investigated the effects of different NO donors on melanoma cell growth and p53 modification. Biotin switch assays demonstrated that treatment with S-nitrosoglutathione (GSNO) or diethylenetriamine (DETA) NONOate significantly increased total S-nitrosylated proteins in A375 and SB2 melanoma cells. p53 was confirmed to undergo SNO under nitrosative stress, as evidenced by biotin switch assays and mass spectrometry. Proteomic analysis identified Cys242, Cys275, and Cys277 as specific SNO sites on p53. Structural modelling revealed that SNO of Cys242 and Cys277 disrupts p53 conformation and impairs its DNA-binding ability. These findings uncover a critical mechanism by which nitrosative stress alters the function of p53 through site-specific SNO, laying a foundation for further exploration of how SNO affects p53-mediated transcriptional regulation in melanoma cells.

## 1. Instruction

Chronic inflammation plays a pivotal role in promoting tumor growth, metastasis, and therapeutic resistance in distinct subsets of human cancers, including melanoma [1]. In the tumor, a chronic inflammatory microenvironment stimulates the generation of reactive oxygen species (ROS) and reactive nitrogen species (RNS), which modulate protein function and disrupt cellular signaling pathways. These reactive species contribute to genomic instability by inducing DNA damage and facilitating the survival and progression of malignant cells [1,2,3,4]. Aberrant expression of nitric oxide synthases (NOSs), particularly inducible NOS (iNOS), leads to sustained production of NO and persistent nitrosative stress. NO is not only a potent mutagenic carcinogen that directly damages deoxyribonucleic acid (DNA) but also a reactive intermediate capable of modifying protein structure and function [5]. Dr. Grimm’s group demonstrated that constitutive expression of iNOS is present in approximately 60% of human metastatic melanoma cases and is significantly associated with poor patient survival [6]. Multiple studies have shown that NO can drive tumor cell proliferation and confer resistance to apoptosis. Notably, pharmacological quenching of NO has been shown to induce G2 phase cell cycle arrest and restore sensitivity to cisplatin-induced apoptosis in melanoma cells [3,6]. These findings underscore the crucial role of aberrant NO production in sustaining chronic nitrosative stress, promoting tumor progression, and mediating therapy resistance in melanoma. Therefore, identifying specific RNS-driven inflammatory markers offers a promising strategy for discovering novel diagnostic biomarkers and developing more effective therapeutic interventions for melanoma.

NO is a pleiotropic signaling molecule that regulates a wide range of physiological and pathological processes, including immune responses, vasodilation, neurotransmission, and cancer progression [7,8]. One of the key mechanisms through which NO exerts its effects is via post-translational modifications (PTMs) of proteins, both reversible and irreversible, thereby influencing protein structure, localization, and function. Among these modifications, SNO, the covalent attachment of a nitroso group to the thiol side chain of cysteine residues, is a unique and increasingly recognized redox-based PTM. SNO has been extensively studied in cardiovascular, neurological, and infectious diseases, where it plays critical roles in regulating cellular homeostasis. However, its role in cancer, particularly in melanoma, remains an emerging and underexplored area of redox biology [8,9,10].

Given the aberrant expression of NOSs and elevated NO levels in melanoma as demonstrated in prior studies [3,6,11], it is plausible that nitrosative stress drives tumorigenesis in part through SNO-mediated modification of key regulatory proteins. Recent studies support this hypothesis. Ding et al. demonstrated that NO exposure induces SNO of the tumor suppressor phosphatase and tensin homolog (PTEN) in melanoma cells, potentially leading to Akt pathway activation and oncogenic signaling [11]. Similarly, Lopez-Rivera et al. reported that SNO of tuberous sclerosis complex 2 (TSC2) impairs its tumor-suppressive function, resulting in hyperactivation of the mTOR pathway and enhanced melanoma cell proliferation [12]. Furthermore, Xu et al. showed that neuronal NOS (nNOS)–mediated SNO of histone deacetylase 2 (HDAC2) disrupts interferon (IFN) signaling, facilitating immune evasion and lung metastasis in melanoma [13]. Although comprehensive and systematic studies of SNO in melanoma are still lacking, accumulating evidence suggests that aberrant SNO of critical proteins contributes to dysfunctional signaling networks and tumor progression. Since SNO can be pharmacologically modulated by targeting nitrosative stress pathways, aberrantly S-nitrosylated proteins represent promising therapeutic targets in melanoma and potentially other cancers characterized by NO dysregulation.

Somatic mutations in the *TP53* gene are relatively rare in cutaneous melanoma, occurring in fewer than 10% of cases, a significantly lower frequency compared to other malignancies such as ovarian, colorectal, and lung cancers, where mutation rates range from 38% to 50% [14,15]. Despite the low prevalence of *TP53* mutations, multiple studies have demonstrated elevated expression and altered function of wild-type (WT) p53 protein in melanoma cell lines and tumors [14,16,17]. Furthermore, amplification and overexpression of the negative regulators of p53, mouse double minute 2 (MDM2) and murine double minute X (MDMX), have been observed in 40%–60% of melanoma cases, suggesting functional suppression of p53 in these tumors [15,18].

Emerging evidence indicates that p53 activity may be modulated by nitrosative stress through conformational changes induced by post-translational modifications [19,20]. Although the precise mechanisms remain unclear, the presence of ten free cysteine residues within the DNA-binding domain of p53, none of which are involved in disulfide bond formation, suggests a high susceptibility to redox-based modifications such as SNO [21,22]. These cysteines exist predominantly as reactive thiolate anions, rendering them particularly sensitive to attack by reactive oxygen and nitrogen species (ROS/RNS) [23]. Several studies have shown that these cysteine residues play a critical role in redox modulation and DNA-binding activity of p53 [19,20]. However, whether endogenous NO in melanoma can induce SNO of p53 and which specific residues are modified, remains unresolved.

In this study, we detected endogenous S-nitrosylated proteins in melanoma cells under conditions of nitrosative stress using a biotin switch assay followed by mass spectrometry. Notably, we confirmed that p53 undergoes SNO in this context, resulting in structural and functional alterations of the protein. These findings provide novel insight into redox-based regulation of wild-type p53 in melanoma and suggest that SNO may represent a mechanism of p53 inactivation in tumors lacking *TP53* mutations.

## 2. Results

### 2.1. Effects of Nitrosative Stress on Melanoma Cell Viability

**Figure 1:**
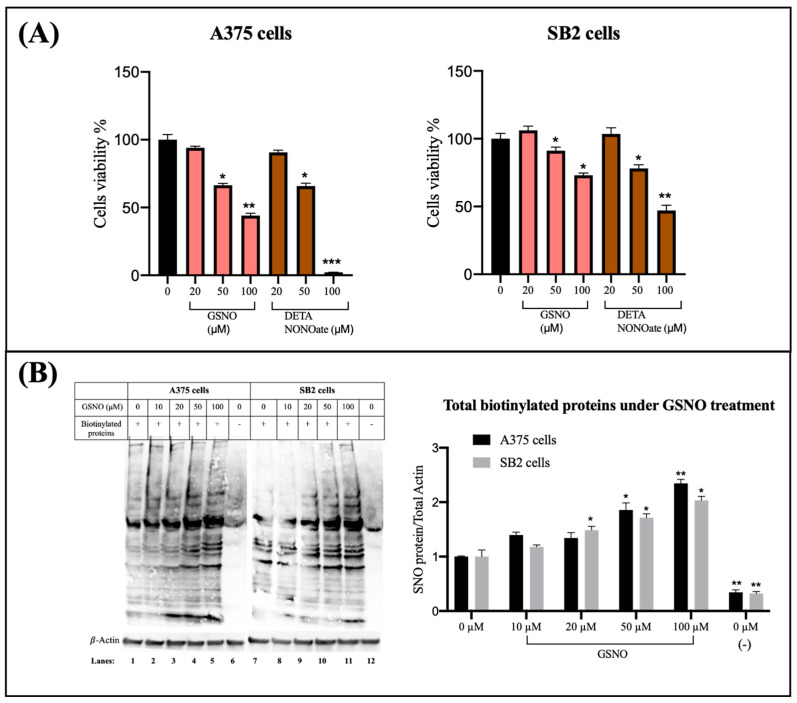
Nitric oxide (NO) donors reduce melanoma cell viability and enhance global protein SNO. (**A**) Cell viability of A375 (BRAF^V600E-mutant) and SB2 (NRAS-mutant) melanoma cells following treatment with the NO donors GSNO and DETA NONOate at concentrations of 20, 50, and 100 µM for 24 h. Cell viability was assessed by MTT assay. Both cell lines exhibited a dose-dependent reduction in viability, with significant cytotoxic effects observed at ≥50 µM concentrations. Data are expressed as a percentage of control (untreated) cells. Pink bars represent the treatment of GSNO, and brown bars represent the treatment of DETA NoNOate. (**B**) Left panel: Representative immunoblot of biotinylated proteins detected by the biotin-switch assay in A375 and SB2 cells treated with GSNO (10, 20, 50, and 100 µM). Non-biotinylated samples (lanes 6 and 12) served as negative controls to confirm the specificity of biotin labeling. β-actin was used as a loading control. Right panel: Quantification of total S-nitrosylated proteins normalized to β-actin expression. Bar graphs represent the mean ± SEM from three independent experiments. Signal intensities for GSNO-treated samples were normalized to untreated controls (0 µM), which were set to 1. * indicate statistically significant differences compared to the control group (* *p* ≤ 0.05; ** *p* ≤ 0.01; *** *p* ≤ 0.001), as determined by one-way ANOVA followed by post hoc analysis.

As shown in Figure 1A, treatment with 20 µM GSNO or DETA NONOate for 72 h had minimal impact on A375 cell viability, with levels comparable to untreated controls. Interestingly, SB2 cells exhibited a modest but statistically significant increase in viability at this lower concentration, suggesting that mild nitrosative stress may transiently promote SB2 cell growth.

In contrast, exposure to higher concentrations (50 and 100 µM) of either NO donor significantly reduced cell viability in both melanoma cell lines. Specifically, A375 cells displayed a dose-dependent decline in viability, with reductions of approximately 40% at 50 µM and over 80% at 100 µM of DETA NONOate (*p* < 0.05 to *p* < 0.001). A similar trend was observed in SB2 cells, which showed viability reductions of 30–50% at higher doses of both NO donors (*p* < 0.05 to *p* < 0.01).

These findings indicate that low-level nitrosative stress does not inhibit, and may even transiently stimulate, melanoma cell growth, whereas high-level nitrosative stress significantly impairs cell viability in a dose-dependent manner.

### 2.2. Nitrosative Stress Induced by GSNO Increases Total S-Nitrosylated Proteins in Melanoma Cells

To evaluate the extent of protein SNO in melanoma cells under nitrosative stress, we performed the biotin-switch assay, which converts labile S-nitrosothiols into stable biotinylated thiols detectable by immunoblotting. A375 and SB2 melanoma cells were treated with increasing concentrations of the NO donor GSNO (10–100 µM), followed by biotin-switch labeling and Western blot analysis.

As shown in Figure 1B (left panel), both A375 and SB2 cells displayed a basal level of biotinylated proteins in untreated samples (lanes 1 and 7), suggesting constitutive SNO under basal culture conditions. These signals were markedly reduced in negative control samples processed without the biotinylating reagent biotin-HPDP (lanes 6 and 12), confirming the specificity of the assay and excluding nonspecific background or contamination.

Upon GSNO treatment, there was a clear and dose-dependent increase in the abundance of biotinylated proteins in both cell lines (lanes 2–5 for A375 and lanes 8–11 for SB2). Notably, exposure to 50 µM and 100 µM GSNO resulted in a substantial elevation of S-nitrosylated protein levels, as evidenced by the intensified banding patterns across a broad molecular weight range.

This trend was quantitatively supported by densitometric analysis normalized to β-actin (Figure 1B, right panel). Treatment with 10 µM GSNO induced a modest increase in S-nitrosylated proteins (approximately 20–40%) in both A375 and SB2 cells. At 100 µM GSNO, the total S-nitrosylated protein levels increased more than two-fold relative to untreated controls, reaching statistical significance (*p* < 0.05 and *p* < 0.01), indicating a robust nitrosative response.

Together, these data demonstrate that GSNO induces a concentration-dependent increase in global protein SNO in melanoma cells. This result underscores the potential of nitrosative stress to modulate cellular signaling through widespread SNO and highlights the utility of the biotin-switch assay for detecting dynamic changes in SNO-modified proteomes under physiological and stress conditions.

### 2.3. p53 Is S-Nitrosylated in Melanoma Cells Under Nitrosative Stress

Given the critical role of p53 as a tumor suppressor that governs cell cycle arrest, DNA repair, and apoptosis, we investigated whether p53 undergoes SNO in melanoma cells subjected to nitrosative stress. A375 and SB2 cells were treated with increasing concentrations of two NO donors, DETA NONOate and GSNO (20, 50, and 100 µM). Following treatment, protein lysates were subjected to the biotin-switch assay, and S-nitrosylated proteins were selectively enriched using streptavidin pulldown, followed by immunoblotting for p53.

**Figure 2:**
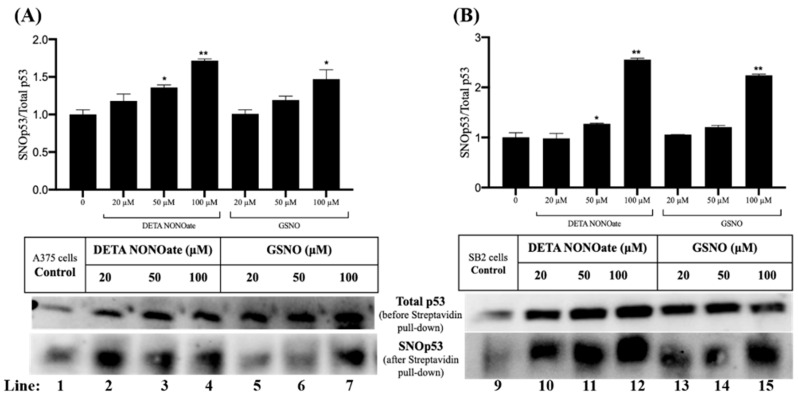
Nitrosative stress enhances p53 SNO in melanoma cells. (**A**) A375 cells and (**B**) SB2 cells were treated with nitric oxide (NO) donors, GSNO, and DETA NONOate, at concentrations of 20, 50, and 100 µM for 24 h. Total cellular lysates were subjected to the biotin-switch assay to detect S-nitrosylated p53 (SNO-p53). The bottom panels show representative Western blots of total p53 (before streptavidin pulldown) and SNO-p53 (after streptavidin pulldown) under each treatment condition. p53 was consistently detected in all samples, confirming both expression and successful SNO detection. The upper panels present densitometric quantification of SNO-p53 levels normalized to total p53. Signal intensity ratios of SNO-p53 normalized to total p53 in untreated control samples (0 µM) were set to 1, and all other conditions were expressed relative to this baseline. The relative SNO-p53 signal intensity in untreated control samples was set to 1. A dose-dependent increase in SNO-p53 was observed in both cell lines, with maximal SNO occurring at 100 µM for both GSNO and DETA NONOate. Bar graphs represent the mean ± SEM from at least three independent experiments. Statistical significance was determined by one-way ANOVA with post hoc comparisons: * *p* ≤ 0.05; ** *p* ≤ 0.01 versus untreated controls.

As shown in the lower panels of Figure 2A,B, total p53 was consistently detected across all treatment conditions prior to streptavidin enrichment, confirming stable p53 expression. Importantly, p53 was also detected in the streptavidin-enriched fractions following the biotin-switch procedure (lanes 1–7 for A375 cells and 9–15 for SB2 cells), indicating that a fraction of p53 exists in a S-nitrosylated form (SNO-p53). Notably, SNO-p53 was also detected under control conditions (lanes 1 and 9), suggesting basal levels of endogenous p53 SNO in both melanoma cell lines, even in the absence of exogenous NO donors.

Treatment with both GSNO and DETA NONOate led to a dose-dependent increase in the relative level of SNO-p53 compared to total p53 (Figure 2). In A375 cells (Figure 2A), 100 µM DETA NONOate induced the highest level of SNO-p53, with an approximate 80% increase relative to control (lane 4; *p* < 0.01), while 100 µM GSNO resulted in a 60% increase (lane 7; *p* < 0.05). Similarly, in SB2 cells (Figure 2B), a pronounced elevation in SNO-p53 was observed with 100 µM of both DETA NONOate and GSNO, with increases of approximately 150% and 130%, respectively (lanes 12 and 15; *p* < 0.01).

These findings confirm that p53 is a bona fide target of SNO in melanoma cells and that nitrosative stress significantly enhances this post-translational modification. Given p53’s central role in stress response and tumor suppression, the SNO of p53 may represent a regulatory mechanism by which nitric oxide modulates p53 function and potentially contributes to melanoma progression or therapeutic resistance. Further investigation is warranted to determine the functional consequences of p53 SNO in the context of melanoma pathobiology.

### 2.4. Identification of S-Nitrosylated Cysteine Residues on p53 in Melanoma Cells Under Nitrosative Stress

The direct and site-specific identification of S-proteins in biological systems remains a significant analytical challenge due to the labile nature of the S-NO bond and the typically low abundance of endogenously S-nitrosylated species. To address this, we utilized an integrated approach combining the biotin-switch assay with high-resolution liquid chromatography-tandem mass spectrometry (LC-MS/MS), which has emerged as a powerful tool for characterizing protein SNO in complex cellular environments.

As shown in previous proteomic analyses of SNO in real biological systems with in vitro NO donor treatments, high concentrations of NO donors (100 µM–10 mM) were used to induce high levels of S-nitrosylated proteins to be detected by MS [24,25]. Therefore, in our study, A375 melanoma cells were treated with 100 µM GSNO for 24 h to ensure sufficient SNO-p53 enrichment for downstream proteomic analysis (Figure 1B). Cell lysates were subjected to the biotin switch assay, and biotinylated proteins were separated by native PAGE. Protein bands migrating near the molecular weight of p53 (~53 kDa) were visualized via Coomassie staining, excised from the gel, digested with trypsin, and analyzed by LC-MS/MS in collaboration with the Proteomics Core Facility at UTHealth Houston.

**Figure 3:**
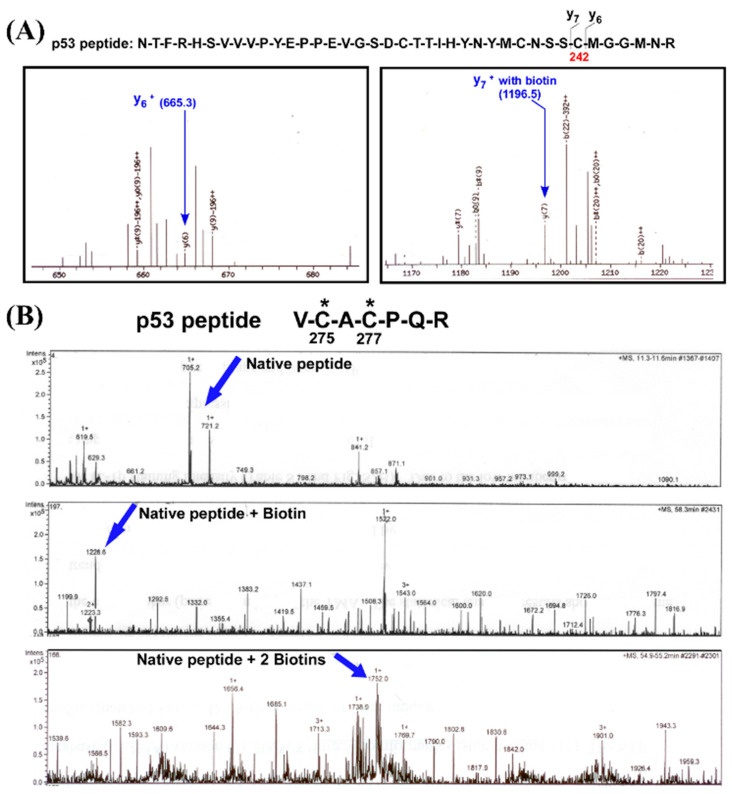
Mass spectrometry (MS) analysis identifies site-specific SNO of p53 in melanoma cells under nitrosative stress. A375 melanoma cells were treated with 100 µM GSNO, and total cellular proteins were subjected to the biotin-switch assay followed by tryptic digestion and LC-MS/MS analysis to identify S-nitrosylated (SNO) residues on p53. The proteomic study identified the SNO profile in melanoma cells under nitrosative stress. (**A**) Tandem mass spectra of the p53 peptide sequence *NTFRHSVVVPYEPPEVGSDCTTIHYNYMCNSSCMGGMNR* revealed biotinylation at Cys242. The left panel shows the detection of the y_6_^+^ ion at 665.3 *m*/*z*, corresponding to the unmodified peptide fragment, while the right panel shows a shift to 1196.5 *m*/*z* (y_7_^+^), indicating the presence of a biotin-labeled Cys242. This confirms SNO at this site following conversion to a stable biotin adduct via the biotin-switch assay. (**B**) MS analysis of a second p53 tryptic peptide, *VCACPQR*, identified additional SNO sites at Cys275 and Cys277. The top panel displays the spectrum of the native peptide; the middle panel shows a single biotinylated species (mono-SNO), and the bottom panel demonstrates a mass shift consistent with the presence of two biotin adducts (di-SNO), confirming that both Cys275 and Cys277 are S-nitrosylated in response to GSNO treatment. These results provide direct proteomic evidence that Cys242, Cys275, and Cys277 are key SNO targets on p53 under nitrosative stress, potentially altering its structural conformation and DNA-binding function in melanoma cells.

As shown in Figure 3A, tandem MS spectra identified a tryptic peptide of p53 (residues 221–242: N-T-F-R-H-S-V-V-V-P-Y-E-P-P-E-V-G-S-D-C-T-T-I-H-Y-N-Y-M-C-S-C-M-G-G-M-N-R) containing Cys242, modified with a biotin adduct indicative of prior SNO. The mass shift corresponding to the y_7_ ion (1196.5 *m*/*z*) confirmed the presence of biotin on this residue. Additionally, in Figure 3B, another p53-derived peptide (residues 275–280: V-C-A-C-P-Q-R) exhibited mono- and di-biotinylated species, revealing two additional sites of SNO at Cys275 and Cys277, respectively. The progressive mass shifts and fragmentation patterns aligned with the expected modifications resulting from maleimide-PEO_2_-biotin derivatization during the biotin switch assay, affirming the specificity of the detection.

Together, these data provide the first direct experimental evidence that Cys242, Cys275, and Cys277 of p53 are targets of SNO in melanoma cells under nitrosative stress. The identification of these modification sites is significant, as all three cysteine residues lie within functionally important domains of p53 that regulate DNA binding and structural conformation. SNO at these sites may, therefore, alter p53 stability, localization, or transcriptional activity, potentially impacting its tumor suppressor functions in the context of the melanoma microenvironment. These findings establish a foundation for future mechanistic studies into how redox modifications fine-tune p53 activity in cancer progression and therapeutic response.

### 2.5. Molecular Dynamics (MD) Simulations Reveal S-Nitrosylation-Induced Conformational Disruption of p53-DNA Binding and Zinc Coordination

To investigate the structural and functional consequences of p53 S-nitrosylation on its DNA-binding and conformational stability, molecular dynamics (MD) simulations were performed on both native and S-nitrosylated forms of p53 bound to the p21 promoter DNA. Simulations were conducted for 100 nanoseconds (ns) using high-resolution crystal structures of the p53-DNA complex as a starting model. Particular focus was placed on residues known to mediate sequence-specific DNA recognition and structural integrity [26].

**Figure 4:**
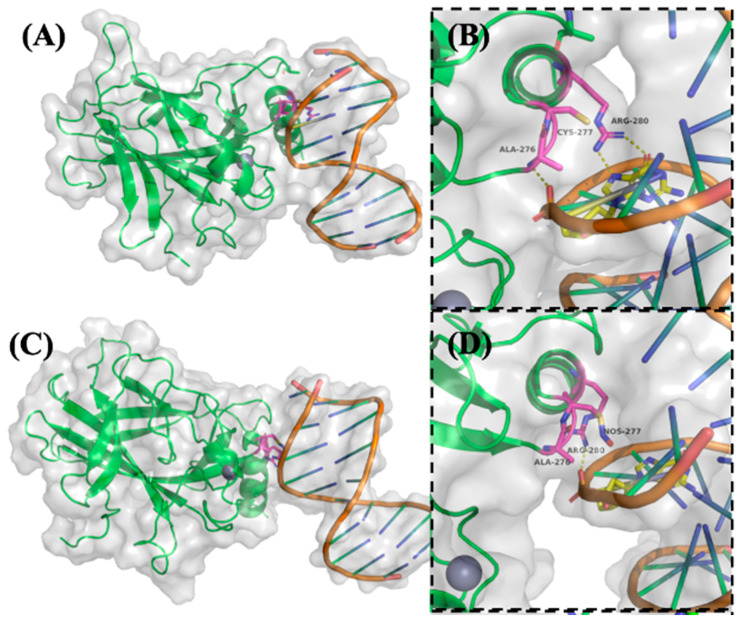
Molecular dynamics (MD) simulation reveals structural disruption of p53–DNA interaction upon SNO. Molecular dynamics (MD) simulation of p53–DNA under SNO modification. (**A**) Crystal structure of the wild-type p53 DNA-binding domain (green) in complex with a double-stranded p21 promoter DNA (orange), showing canonical binding conformation (PDB ID: 2AHI). (**B**) Zoomed-in view highlighting key DNA contact residues: Arg280 forms hydrogen bonds with a guanine base in the DNA major groove, and Ala276 forms a hydrogen bond with the DNA phosphate backbone (yellow dashed lines), stabilizing the p53–DNA interface. (**C**) MD-simulated model of S-nitrosylated p53 (SNO-p53) bound to DNA, incorporating SNO modifications at Cys242, Cys275, and Cys277. (**D**) Structural overlay illustrating the conformational consequences of Cys277 SNO (pink). The bulky SNO group induces displacement of Arg280 away from the DNA major groove, disrupting its guanine contacts and abolishing the hydrogen bond between Ala276 and the DNA backbone. Non-modified p53 is shown in green/purple; S-nitrosylated p53 in blue/orange. Structural visualizations were rendered using PyMOL (https://pymol.org/2/ (accessed on 1 March 2022 to 28 February 2023)). These findings suggest that SNO of Cys277 compromises specific DNA binding, potentially attenuating p53 transcriptional activity.

As shown in Figure 4A,B, native p53 engages DNA via a well-structured interaction interface. Arg280 forms two critical hydrogen bonds with a conserved guanine base in the DNA major groove, serving as a key anchoring residue. Ala276 stabilizes the complex through a hydrogen bond with the DNA phosphate backbone, while Cys277 lies in close proximity, potentially influencing local structural conformation [26].

Upon SNO of Cys277, substantial steric and electrostatic alterations were observed in the simulation trajectory (Figure 4C,D). The addition of the bulky –NO group to Cys277 reorients Arg280 away from the DNA major groove, thereby abolishing its hydrogen bonding with the guanine base. Simultaneously, the original stabilizing interaction between Ala276 and the DNA backbone is lost. A compensatory hydrogen bond between Arg280 and the DNA phosphate backbone emerges, yet this altered configuration suggests a weakened and less specific binding interface. These findings indicate that SNO at Cys277 disrupts the precise spatial arrangement required for optimal DNA recognition, likely impairing the transcriptional activation of p53 target genes.

**Figure 5:**
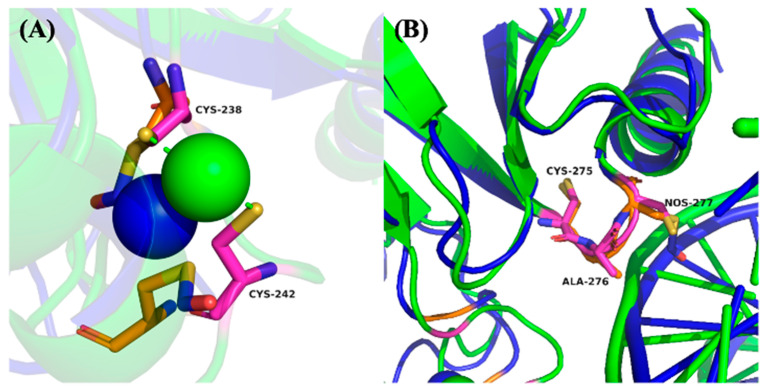
S-nitrosylation of p53 alters zinc coordination and local structural conformation. Overlay of wild-type and S-nitrosylated p53 molecular dynamics simulations. (**A**) Molecular dynamics (MD) simulation of the p53 zinc-binding domain comparing wild-type (WT) and SNO states. Cys238 and Cys242, critical for Zn^2+^ coordination, are shown to interact with the zinc ion. In WT p53, both cysteine residues maintain optimal proximity to the Zn^2+^ ion (green sphere). Upon SNO of Cys242, a significant displacement is observed (Cys242 shown in orange), weakening zinc chelation, with the altered Zn^2+^ position shown as a blue sphere. This disruption may destabilize the structural integrity of the DNA-binding domain. (**B**) MD simulation overlay showing local structural changes surrounding Cys275 and Cys277. While Cys275 (pink) remains conformationally stable with or without SNO, SNO modification of Cys277 (orange) causes spatial reorientation of adjacent residues, including Ala276, potentially altering DNA contact surface topology. Wild-type p53 is depicted in green/purple; S-nitrosylated p53 in blue/orange. These simulations underscore residue-specific structural perturbations caused by SNO, particularly at Cys242 and Cys277, with potential implications for p53 functional inactivation under nitrosative stress.

In addition to DNA binding, p53 activity is critically dependent on zinc ion (Zn^2+^) coordination, which stabilizes the DNA-binding domain. Cys238 and Cys242 directly participate in Zn^2+^ chelation and are essential for maintaining the correct protein fold [27,28]. As illustrated in Figure 5A, SNO of Cys242 induces a noticeable displacement of this residue away from the Zn^2+^ ion, diminishing its chelation capacity and suggesting partial destabilization of the p53 structural core. This conformational shift could reduce the overall stability of the DNA-binding domain, thereby compromising p53’s functional integrity under nitrosative stress conditions.

In contrast, Figure 5B shows that SNO of Cys275, which is located within a hydrophobic cavity distant from the DNA-binding interface, results in minimal conformational fluctuation during the simulation. This suggests that Cys275 SNO may not significantly alter the global structure or DNA-binding activity of p53, highlighting site-specific differences in the functional impact of SNO modifications.

Collectively, these MD simulations provide mechanistic insight into how site-specific SNO disrupts critical DNA interactions and zinc coordination in p53. The observed structural perturbations likely contribute to impaired transcriptional activity and reduced tumor suppressor function of p53 under nitrosative stress—conditions often prevalent in inflammatory tumor microenvironments.

## 3. Discussion

In this study, we present novel mechanistic insights into how nitrosative stress modulates p53 function in melanoma cells through site-specific SNO. By integrating biotin switch assays, mass spectrometry-based SNO-proteomics, and MD simulations, we identified Cys242, Cys275, and Cys277 as critical sites of SNO on p53. These redox modifications disrupt p53’s DNA binding conformation, thereby impairing its transcriptional activity in the absence of genetic mutations. This redox-dependent inactivation reveals a previously underappreciated mechanism by which chronic nitrosative stress in the tumor microenvironment may promote melanoma progression, therapeutic resistance, and immune evasion. Our findings lay the groundwork for further investigation into SNO as a regulatory mechanism for tumor suppressors and underscore its broader relevance in inflammation-associated malignancies.

The tumor microenvironment of metastatic melanoma is marked by chronic inflammation and oxidative stress, both of which promote tumor progression, metastasis, and resistance to therapy [1]. NOSs, particularly iNOS, play a central role in this inflammatory milieu by producing substantial levels of NO, a highly reactive molecule that serves as both a mutagenic oxidant capable of causing DNA damage and a regulator of protein function via post-translational modifications such as SNO [8,10]. SNO is now recognized as a key mechanism by which NO exerts its biological effects, modulating numerous cellular processes, including gene transcription, apoptosis, and signal transduction.

Our findings, along with previous reports [7,29,30], support the dual role of NO in cancer: at low concentrations (pM–nM), NO promotes tumor survival by enhancing anti-apoptotic signaling, whereas at higher concentrations (μM), NO becomes cytotoxic. In melanoma, the constitutive expression of iNOS has been detected in approximately 60% of metastatic patient biopsies and correlates strongly with poor clinical outcomes [6,11]. NO has been shown to promote cell proliferation and confer resistance to apoptosis, while its scavenging can induce G2 arrest and enhance cisplatin-induced apoptosis in cancer cells [3]. These data suggest that endogenous NO at low-to-moderate concentrations may help sustain chronic oxidative stress that fosters tumor cell survival and resistance to treatment. Additionally, NO also contributes to immune evasion in tumors by suppressing anti-tumor immune responses [31,32]. In our study, BRAF(V600E)-mutant A375 cells and NRAS-mutant SB2 cells exhibited minimal cytotoxicity at 20 µM NO donor exposure, while higher doses (50–100 µM) induced approximately 50% cell death (Figure 1A), highlighting the dose-dependent biological response to nitrosative stress.

Despite the wide use of NO donors in preclinical models, it remains technically difficult to precisely quantify intracellular NO levels due to variable cellular uptake and antioxidant buffering. Although micromolar concentrations of NO donors were applied in our study and other reports [10,12,23], the actual bioavailable NO inside the cell is likely to be much lower. Furthermore, detecting endogenous SNO proteins remains a challenge because of their low abundance and the instability of the S-NO bond. We addressed this limitation by using 100 µM GSNO to enhance SNO-p53 levels, enabling successful identification via mass spectrometry. Future investigations should incorporate more sensitive and selective enrichment strategies, such as cysteine thiol–reactive iodoacetyl tandem mass tags (iodoTMT) or advanced fluorescence-based NO probes, to map the S-nitrosoproteome under lower, physiologically relevant nitrosative stress conditions. These approaches will be especially critical for translational studies aiming to profile SNO events in clinical melanoma specimens [33,34,35].

Post-translational modifications (PTMs) are essential regulators of p53 function, modulating its ability to bind DNA and control transcription of downstream targets. p53 contains 10 cysteine residues within its DNA-binding domain, several of which are susceptible to redox modifications under oxidative or nitrosative stress [36,37,38,39,40]. Notably, residues Cys176, Cys182, Cys238, Cys242, Cys275, and Cys277 have been implicated in redox regulation, with modifications often reducing p53’s DNA-binding affinity and altering its transcriptional function [38,39,40]. Our MD simulations reveal that SNO of Cys242 weakens its ability to coordinate Zn^2+^, consistent with prior findings by Scotcher et al., who showed that oxidation of Cys238 and Cys242 results in zinc displacement and structural destabilization of p53 [38]. Moreover, Cys277 has been shown to undergo S-glutathionylation upon exposure to oxidants, further supporting its role as a redox-sensitive residue [38]. Our results confirm that Cys277 is S-nitrosylated in melanoma cells and that this modification significantly alters the spatial positioning of adjacent DNA contact residues, particularly Arg280, thereby compromising specific DNA recognition (Figure 3 and Figure 4).

While mutagenesis studies can elucidate the functional roles of specific residues, cysteine-to-alanine or serine substitutions do not fully recapitulate the structural effects of SNO modifications. Nonetheless, mutations at Cys242, Cys275, and Cys277 have consistently been shown to impair p53 structure or DNA binding [38,39,40,41,42]. For example, mutation of Cys277 to various residues (C277S, C277A, C277D) disrupts sequence-specific DNA recognition and abolishes transcriptional activity at key promoter sites [40,41,42]. Schaefer et al. reported that C275S and C277S mutations impaired p53 dissociation from DNA under oxidative stress, although only C275S reduced affinity for the Gadd45 promoter [39]. These findings align with our structural modeling, which suggests that Cys275 lies within a solvent-accessible cavity not directly involved in DNA contact. Thus, the SNO of Cys275 may not affect p53 binding to the p21 promoter used in our simulations, but it may still influence p53 interactions at alternative response elements. The variability in promoter architecture likely contributes to the differential effects of cysteine modifications on p53 function.

Beyond DNA binding, redox regulation of p53 may also modulate protein–protein interactions. The C242S mutation has been shown to disrupt p53 binding to macrophage migration inhibitory factor (MIF), further emphasizing the broader regulatory implications of redox-sensitive cysteines [43]. This study reinforces the critical role of Cys242, Cys275, and Cys277 in modulating p53’s structural and functional integrity, particularly under conditions of nitrosative stress.

In addition to SNO, disulfide bond formation among free cysteines is a well-established mechanism mediating oxidative regulation of p53 [38,39,44]. Studies in vascular smooth muscle cells (SMCs) have shown that disulfide bonds, rather than SNO, dominate redox signaling responses, with SNO levels highly dependent on intracellular glutathione [44]. Whether melanoma cells recapitulate this regulatory pattern remains unclear, given their diverse genetic and metabolic profiles. In our experiments, we detected both endogenous and inducible SNO modifications of p53, suggesting that melanoma cells possess a substantial capacity for SNO under nitrosative stress. Further investigation is warranted to quantify total disulfide formation and glutathione levels in melanoma, especially within the tumor microenvironment.

**Figure 6:**
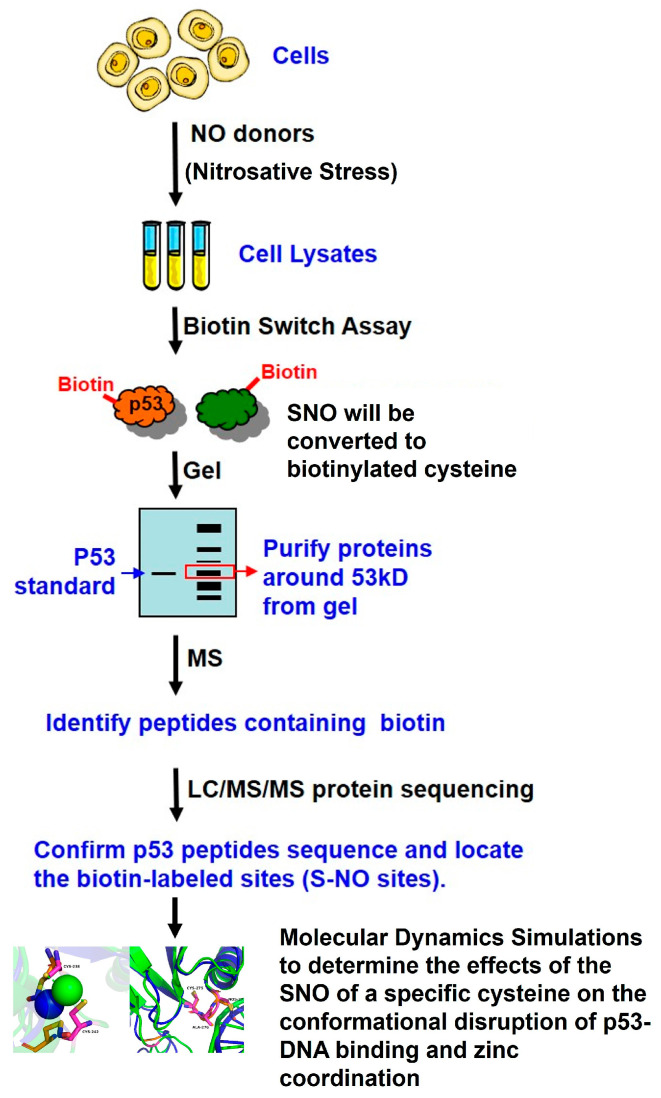
Graphical summary of the experimental workflow being used to identify S-nitrosylated p53 in melanoma cells under nitrosative stress.

Although the full spectrum of redox regulatory mechanisms in cancer remains to be elucidated, SNO clearly represents a dynamic and functionally relevant PTM that modulates key tumor suppressor functions, such as p53, based on this study and other reports in other cancers with consistent findings [19,20]. In glioma cells, peroxynitrite was shown to impair p53 transcriptional activity by preventing DNA binding, resulting in p53 accumulation without functional output [20]. Our data support a similar mechanism in melanoma, demonstrating that SNO inhibits p53–DNA interaction by altering both binding conformation and zinc coordination (Figure 5 and Figure 6).

Ongoing studies in our laboratory are investigating the functional consequences of p53 SNO using a multifaceted experimental approach. We are currently employing electrophoretic mobility shift assays (EMSA) and fluorescence-based DNA-binding assays to evaluate how SNO affects p53’s ability to bind its consensus DNA motifs. In parallel, we are integrating chromatin immunoprecipitation sequencing (ChIP-seq), site-directed mutagenesis of S-nitrosylation-sensitive cysteine residues, and transcriptomic profiling under nitrosative stress conditions to elucidate how SNO modulates p53 target gene regulation in both physiological and tumorigenic contexts.

In our preliminary EMSA results (Supplementary Figure S3), nuclear extracts from untreated A375 melanoma cells formed strong DNA–protein complexes with the conserved human p53-consensus-binding DNA motif, while extracts from cells treated with 100 µM DETA NONOate exhibited reduced complex formation. This suggests that nitrosative stress impairs the DNA-binding activity of p53 in melanoma cells. To further understand downstream functional effects, we analyzed the expression of well-known downstream genes regulated by p53, including p21, MDM2, and PUMA, following treatment with increasing concentrations (20, 50, and 100 µM) of the NO donors DETA NONOate and GSNO (Supplementary Figure S4). Both agents significantly upregulated p53, p21, and MDM2 expression in a dose-dependent manner in A375 and SB2 cells (*p* ≤ 0.05). Interestingly, PUMA expression showed a cell line-dependent response: while A375 cells exhibited no significant change, SB2 cells demonstrated a marked reduction in PUMA levels at higher NO donor concentrations (50 and 100 µM), indicating selective repression of pro-apoptotic signaling under nitrosative stress. These findings support the hypothesis that SNO modulates p53 transcriptional activity in a gene-specific and cell context-dependent manner, potentially influencing melanoma cell fate decisions. To translate these findings in vivo, we initiated efforts to establish a cohort of melanoma tumor samples stratified by nitrosative stress levels. As a proxy for SNO, we performed IHC using anti-nitrotyrosine antibodies to identify tumors with elevated nitrosative stress (Supplementary Figure S5). Given the labile and unstable nature of S-nitrosothiol modifications, ongoing efforts focus on collecting large quantities of fresh–frozen melanoma tumors for future biotin-switch assays and mass spectrometry-based detection of p53 SNO in clinical specimens. These comprehensive studies are currently in progress.

Although all cells in our *in vitro* experiments were uniformly exposed to nitric oxide (NO) donors via the culture medium, a potential limitation of this approach is that the contribution of NO-mediated bystander effects was not directly assessed. Given the high diffusibility of NO and its reactive derivatives, these molecules can traverse cell membranes and potentially influence neighboring cells that may not have been directly targeted by the initial NO donor. This type of indirect cellular response, commonly referred to as the bystander effect, has been reported in multiple biological systems, including radiation exposure, immune signaling, and redox biology [45]. In our experimental conditions, while the entire cell population received NO donor treatment, it remains possible that NO released by individual cells further propagated redox signals to adjacent cells, potentially amplifying or altering p53 SNO in a non-cell-autonomous fashion. Although this was not the focus of the current study, we acknowledge this limitation. Future investigations using spatially resolved models, such as co-culture systems or microfluidic devices, would be valuable to delineate localized versus systemic NO signaling and better understand the scope of bystander effects in melanoma and related tumor contexts.

Moreover, SNO may intersect with other redox-regulated death pathways, such as ferroptosis, an iron-dependent, non-apoptotic mechanism driven by lipid peroxidation [46,47,48]. Recent studies suggest that increased NO levels can trigger ferroptosis in certain cancer models [49,50,51,52,53,54]. Thus, nitrosative stress may not only inactivate tumor suppressors like p53 via SNO but also initiate ferroptotic death. A systems-level analysis integrating SNO proteomics, ferroptosis markers, and transcriptional profiling will be essential for elucidating how redox signaling dictates cancer cell fate in melanoma and beyond.

SNO is an important redox-dependent post-translational modification that regulates protein function in response to nitrosative stress and has growing relevance in cancer biology. Recent advances in SNO-proteomics have uncovered numerous SNO proteins that are associated with tumor progression, immune modulation, and therapeutic resistance, particularly in cancers such as pancreatic and lung carcinomas [55,56]. In the context of melanoma, where chronic nitrosative stress is a well-documented feature of the tumor microenvironment, our study reveals that site-specific SNO of p53 may contribute to tumor progression by functionally disabling this critical tumor suppressor. This redox-based inactivation of p53 may also help explain mechanisms underlying resistance to therapy and immune escape, thereby providing a new dimension to our understanding of redox regulation in cancer.

Importantly, the implications of SNO-mediated modulation of p53 extend beyond melanoma. Other inflammation-driven malignancies, including colorectal and breast cancers, also exhibit elevated nitric oxide levels and oxidative stress signatures. Our mechanistic findings thus offer a broader framework for exploring SNO p53 and related redox-sensitive proteins as candidate biomarkers and therapeutic targets across a spectrum of inflammation-associated tumors.

Furthermore, several NOS inhibitors, particularly those targeting iNOS, have shown therapeutic promise in preclinical tumor models by limiting nitrosative stress and its downstream effects [57]. However, despite these encouraging results, none of these agents have yet advanced into clinical trials for colorectal or other solid tumors, underscoring a significant translational gap. Our study provides mechanistic evidence that supports the functional importance of nitrosative stress in regulating key tumor suppressors like p53 and establishes a conceptual platform for future research aimed at delineating the complex dynamics of redox signaling in cancer. These insights could inform the development of novel therapeutic strategies aimed at modulating SNO or targeting nitrosative stress to restore tumor suppressor function and enhance treatment efficacy in inflammatory tumor types.

## 4. Materials and Methods

### 4.1. Melanoma Cell Culture

This study utilized two human melanoma cell lines: the BRAF^V600E mutant A375 and the NRAS mutant SB2. A375 cells, derived from a metastatic melanoma lesion, were obtained from the American Type Culture Collection (ATCC), while SB2 cells, originating from a primary melanoma, were generously provided by Dr. Elizabeth Grimm at The University of Texas MD Anderson Cancer Center (Houston, TX, USA). Both A375 and SB2 cell lines express wild-type *TP53*, as confirmed by previously published studies and validated by our group [58].

To ensure the authenticity of the melanoma cell lines, short tandem repeat (STR) DNA profiling was performed using the AmpF/STR Identifiler PCR Amplification Kit (Applied Biosystems, Waltham, MA, USA), in accordance with the manufacturer’s instructions. STR analysis was conducted annually by the Characterized Cell Line Core Facility at The University of Texas MD Anderson Cancer Center. In addition, all cell lines were routinely tested for mycoplasma contamination using the MycoStrip^®^ mycoplasma detection assay (InvivoGen, San Diego, CA, USA) every three months to confirm they were mycoplasma-free. All experiments were carried out using cells within 15 passages from early stocks (passages 3–4) directly expanded from the original vials obtained from ATCC or Dr. Grimm’s laboratory.

Cells were cultured in Dulbecco’s Modified Eagle Medium (DMEM; Gibco, NY, USA) supplemented with 10% fetal bovine serum (FBS), 2 mM L-glutamine, 1% HEPES buffer, 100 U/mL penicillin, and 100 µg/mL streptomycin. Cultures were maintained at 37 °C in a humidified incubator with 5% CO_2_.

### 4.2. Antibodies and Reagents

The nitric oxide donor diethylenetriamine NONOate (DETA-NONOate) was purchased from Cayman Chemical (Ann Arbor, MI, USA) and prepared as a 100 mM stock solution in phosphate-buffered saline (PBS). S-nitrosoglutathione (GSNO) was obtained from Tocris Bioscience (Bristol, UK) (CAS No. 57564-91-7) and also dissolved in PBS to a final stock concentration of 100 mM. 3-(4,5-Dimethylthiazol-2-yl)-2,5-diphenyltetrazolium bromide (MTT) reagent was purchased from Alfa Aesar (Ward Hill, MA, USA)(Cat. No. T13G035) and prepared as a 5 mg/mL stock solution in PBS.

Primary antibodies used in this study included anti-p53 (Active Motif, Carlsbad, CA, USA) and anti-β-actin (R&D Systems, Minneapolis, MN, USA). The following horseradish peroxidase (HRP)-conjugated secondary antibodies were used: mouse anti-rabbit IgG-HRP (Santa Cruz Biotechnology, Dallas, TX, USA) and mouse IgG Fc binding protein-HRP (m-IgG Fc BP-HRP; Santa Cruz Biotechnology, Dallas, TX, USA). The specificity of the anti-p53 antibody was confirmed through siRNA-mediated p53 knockdown, as shown in Supplementary Figure S1.

To ensure experimental consistency and reproducibility, all key reagents and antibodies were used from the same batch throughout the duration of the study.

### 4.3. MTT Assay

Cell viability was assessed using the MTT assay. A375 and SB2 melanoma cells were seeded at a density of 10,000 cells per well in 24-well plates and allowed to adhere overnight. Cells were then treated for 72 h with NO donors, either DETA-NONOate or S-nitrosoglutathione (GSNO), at concentrations of 20, 50, or 100 µM. To maintain consistent exposure, the culture medium was replaced every 24 h with a fresh medium containing newly prepared NO donors. Following the 72-h treatment period, 20 μL of MTT reagent (5 mg/mL in PBS) was added to each well, and plates were incubated at 37 °C for 3 h. After incubation, the medium was carefully removed, and 200 μL of dimethyl sulfoxide (DMSO) was added to each well to dissolve the formazan crystals. The absorbance was measured at 570 nm and 630 nm using a BioTek Synergy H1 microplate reader (Santa Clara, CA, USA). Cell viability was calculated as a percentage relative to untreated control wells.

### 4.4. Biotin Switch and Streptavidin-Agarose Pull-Down Assays

A total of 1 × 10^6^ A375 or SB2 melanoma cells were seeded into 100 mm × 20 mm tissue culture dishes and allowed to attach for 24 h. Cells were then treated with DETA-NONOate or GSNO at concentrations of 20, 50, or 100 µM for 72 h, with the treatment medium refreshed every 24 h to ensure sustained nitrosative conditions. Cells were harvested, and total protein lysates were prepared for the biotin switch assay, following the protocol provided with the S-Nitrosylated Protein Detection Kit (Cayman Chemical, Ann Arbor, MI, USA). In brief, all free thiol groups on proteins were first blocked. Next, S-nitrosylated cysteine residues were selectively reduced to free thiols, which were subsequently labeled with biotin. The extent of SNO was assessed using a streptavidin-agarose pull-down assay and confirmed by Western blot analysis for specific target proteins.

To improve assay specificity and minimize false-positive signals, especially those arising from ascorbate overreaction, we introduced a refined negative control. These samples omitted the biotin-HPDP labeling reagent but retained the ascorbate reduction step. This approach distinguished endogenous biotin-containing proteins from artificially biotinylated proteins. Control results closely paralleled those from traditional non-ascorbate-treated samples (Supplementary Figure S2), enhancing the assay’s reliability.

For streptavidin-based enrichment, 100 µg of biotin-labeled protein was diluted in 500 µL of SNO wash buffer (Cayman Chemical, Ann Arbor, MI, USA) and incubated with 70 µL of pre-washed high-capacity streptavidin agarose resin (UBPBio, Pittsburgh, PA, USA) for 1 h at room temperature. The resin was pelleted by centrifugation, and the supernatant was discarded. Pellets were washed three times with 1× PBS. To elute the bound biotinylated proteins, 50 µL of Laemmli sample buffer (bioPLUS Chemicals, Gujarat, India) containing 5% β-mercaptoethanol (Acros Organics, Geel, Belgium) was added to each pellet, followed by boiling at 100 °C for 10 min. The samples were centrifuged, and the supernatants containing enriched S-nitrosylated proteins were collected for subsequent molecular analyses, including immunoblotting.

### 4.5. Western Blot (WB) Assay

Cells were lysed in a buffer containing 50 mM Tris (pH 7.9), 1% Triton X-100, 50 mM HEPES, 150 mM NaCl, 1.5 mM MgCl_2_, 1 mM EGTA, 100 mM NaF, 10 mM sodium pyrophosphate (AlNaO_7_P_2_), 1 mM sodium orthovanadate (Na_3_VO_4_), 10% glycerol, and a protease inhibitor cocktail (Roche, Indianapolis, IN, USA). Protein concentrations were determined, and equal amounts of protein were separated by SDS-PAGE on 4–15% gradient polyacrylamide gels. Following electrophoresis, proteins were transferred onto Hybond ECL nitrocellulose membranes (GE Healthcare Biosciences, Piscataway, NJ, USA). Membranes were blocked with 4% bovine serum albumin (BSA) in PBS for 1 h at room temperature, followed by incubation with primary antibodies overnight at 4 °C. After washing, membranes were incubated with appropriate HRP-conjugated secondary antibodies. Protein signals were detected using an enhanced chemiluminescence (ECL) substrate (GE Healthcare Biosciences, Chicago, IL, USA) and visualized with the ChemiDoc MP Imaging System (Bio-Rad, Hercules, California, USA).

Densitometric analysis was performed using NIH ImageJ software (version 1.50i, Bethesda, MD, USA). For Figure 1B, the densitometric quantification of total S-nitrosylated proteins was normalized to β-actin expression. Bar graphs represent the mean ± SEM from three independent experiments. Signal intensities for GSNO-treated samples were normalized to untreated controls (0 µM), which were set to 1. For Figure 1, the upper panels present densitometric quantification of SNO-p53 levels normalized to total p53. Signal intensity ratios of SNO-p53 normalized to total p53 in untreated control samples (0 µM) were set to 1, and all other conditions were expressed relative to this baseline.

### 4.6. Mass Spectrometry (MS) Protein Sequencing to Identify SNO Sites of p53

To identify S-nitrosylated sites on p53, we employed a combined approach using the biotin switch assay and LC-MS/MS, performed in collaboration with the Proteomics Core Facility at the Brown Foundation Institute of Molecular Medicine, University of Texas Health Science Center at Houston.

A375 melanoma cells treated with NO donors were subjected to the biotin switch assay using the Cayman S-Nitrosylated Protein Detection Kit (Item No. 10006518). Biotin-labeled proteins were separated by native PAGE, and protein bands around 53 kDa were visualized via Coomassie Brilliant Blue staining. The relevant gel slices were excised, destained, and digested with trypsin according to established protocols [59]. Peptides were extracted and analyzed by LC-MS/MS on an LTQ-Orbitrap XL mass spectrometer (Thermo Fisher Scientific, Waltham, MA, USA) coupled with an Eksigent NanoLC-Ultra 2D Plus CHiPLC Nanoflex system (AB SCIEX). Chromatographic separation was performed on a ChromXP C18-CL column. Raw data files were converted to Mascot Generic Format using Mascot Distiller and searched against the SwissProt_2012_01 (Human) database using Mascot v2.3.02 (Matrix Science). Spectra were also matched against a decoy database with false discovery rates (FDRs) set at 1% (strict) and 5% (relaxed). Based on the Cayman kit chemistry, S-nitrosylated cysteine residues were detected as maleimide-PEO_2_-biotin adducts.

All proteomic data were deposited in the ProteomeXchange Consortium with the dataset identifier PXD031807, titled *S-nitrosylation of p53 in A375 Cells*.

### 4.7. Molecular Dynamics (MD) Simulation

Molecular dynamics simulations were conducted using Schrödinger software (2018-1). Among the available crystal structures of the p53-DNA complex, PDB entry 2AHI (with the DNA sequence CGGACATGTCCG) was selected for its high similarity to the p21 response element (GAACATGTCCCAACATGTT). For computational efficiency, only chain A of the p53 tetramer bound to DNA was used in the simulation. Chain A contains all critical amino acid residues involved in DNA binding and zinc (Zn^2+^) coordination, thereby preserving the essential conformation of the DNA-binding domain of p53. The structure was placed in an orthorhombic water box with a 10 Å buffer, and counterions were added to neutralize the system. The system was first equilibrated using a short NVT (constant volume and temperature) simulation followed by an NPT (constant pressure and temperature) simulation. Production MD was then performed for 100 ns in the NPT ensemble at 27 °C using the Nose–Hoover chain thermostat. The simulation used a 2 fs timestep, with trajectories saved every 100 ps. The OPLS3e force field was applied, and the water molecules were modeled using the simple point charge (SPC) method. Electrostatic interactions were handled using a 9 Å cutoff.

The experimental workflow and study design used to confirm p53 SNO under nitrosative stress and to identify SNO-modified sites via mass spectrometry are summarized in Figure 6.

### 4.8. Statistical Analysis

All quantitative data are expressed as the mean ± standard error of the mean (SEM) from at least three independent experiments. Statistical significance between groups was evaluated using two-way analysis of variance (ANOVA), followed by Bonferroni-corrected Tukey’s post hoc test, implemented in GraphPad Prism software (version 9.0). A *p*-value less than 0.05 was considered statistically significant.

## 5. Conclusions

This study provides the first mechanistic insight into how p53 is functionally inactivated through site-specific SNO in melanoma cells under nitrosative stress. Using an integrated approach combining SNO-proteomics and molecular dynamics simulations, we identified Cys242, Cys275, and Cys277 as key SNO sites that disrupt p53’s DNA-binding conformation and impair its transcriptional activity. These findings reveal a redox-dependent mechanism of p53 inactivation that operates independently of genetic mutation, potentially contributing to melanoma progression, therapy resistance, and immune evasion. Given the prevalence of nitrosative stress in the tumor microenvironment of various cancers, including colorectal and breast malignancies, our results offer a conceptual framework for investigating S-nitrosylated p53 and related redox-sensitive proteins as candidate biomarkers and therapeutic targets in inflammation-associated cancers. Future studies will be directed toward validating these mechanisms in vivo and exploring their clinical relevance across tumor types.

## Data Availability

The proteomic data were uploaded to the repository ProteomeXchange under the Project Name S-nitrosylation of p53 in A375 cells (https://www.proteomexchange.org/, project accession: PXD031807).

## Supplementary Materials

The following supporting information can be downloaded at: https://www.mdpi.com/article/10.3390/ijms26136512/s1.
