# Supplementary material for: S-Nitrosylation of p53 in Melanoma Cells Under Nitrosative Stress
Source: Int J Mol Sci. 2025 Jul 6;26(13):6512. doi: 10.3390/ijms26136512 (PMC12250093; doi:10.3390/ijms26136512)
Supplement: Supplementary file 1 [file ijms-26-06512-s001.zip › ijms-3689773-supplementary.pdf]

1. Validation of anti-p53 antibody specificity by siRNA-mediated knockdown in A375 melanoma cells

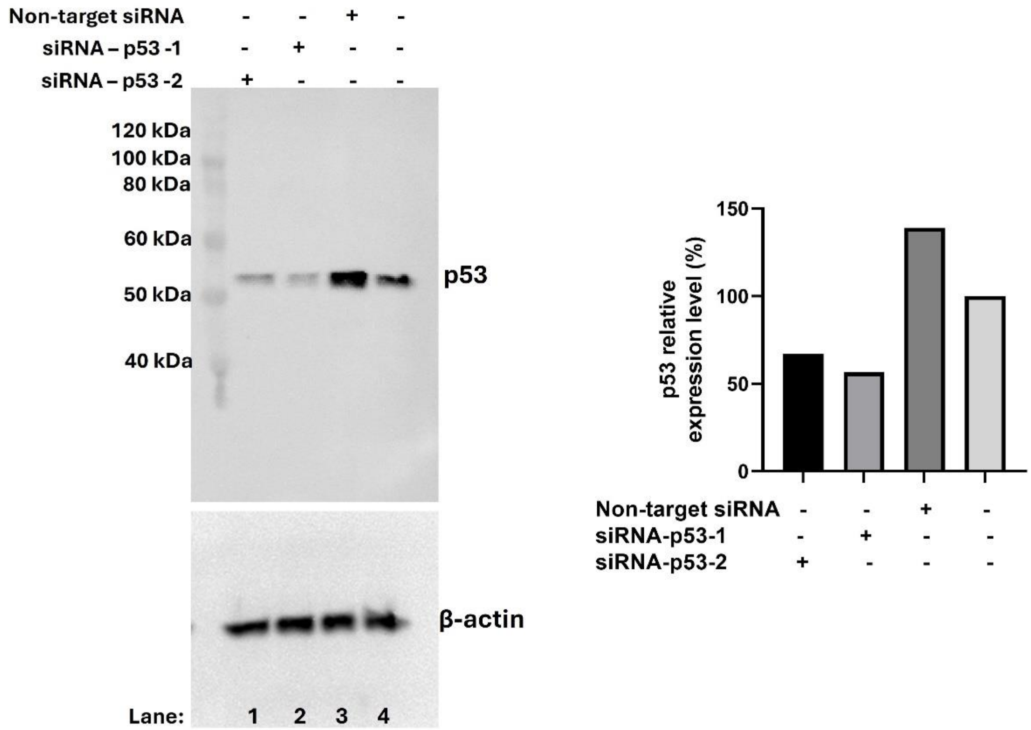

Supplementary Figure S1. Validation of anti-p53 antibody specificity by siRNA-mediated knockdown in A375 melanoma cells. A375 melanoma cells were transfected with 20 nM of either human p53-specific siRNA (siRNA-p53-1 or siRNA-p53-2) or a non-targeting negative control siRNA using Lipofectamine RNAiMAX, following the manufacturer's instructions. After 24 hours, cells were harvested, and protein lysates were analyzed by Western blot using anti-p53 (Active Motif, Cat. No. 39553; ~53 kDa) and anti-β-actin (R&D Systems, Cat. No. MAB8929; ~45 kDa) antibodies. Molecular weight markers were included to verify the expected migration of target proteins. Both p53-targeting siRNAs effectively reduced p53 protein expression compared to the non-targeting control, confirming the specificity of the anti-p53 antibody. β-actin served as a loading control to ensure equal protein input across all lanes.

The right panel displays densitometric quantification of p53 protein levels normalized to β-actin. Signal intensity in the untreated control group (Lane 4) was set to 1, and relative expression levels in all other conditions were calculated accordingly. This analysis supports the reproducibility and specificity of p53 detection under the experimental conditions used. Additionally, in related SNO studies, densitometric quantification of SNO-p53 levels was normalized to total p53, allowing assessment of redox-modified p53 relative to total protein levels.

Result

To confirm the specificity of the anti-p53 antibody used in our Western blot analyses, we performed siRNA-mediated knockdown of p53 in A375 melanoma cells using two distinct p53-targeting siRNAs (siRNA-p53-1 and siRNA-p53-2). Cells were transfected with 20 nM siRNA and harvested 24 hours post-transfection for protein analysis. As shown in Supplementary Figure 1, Western blotting with the anti-p53 antibody (Active Motif, Cat. No. 39553) revealed a marked reduction in p53 protein levels in both siRNA-treated groups compared to cells transfected with non-targeting control siRNA. β-actin

(R&D Systems, Cat. No. MAB8929) and cells were not treated with any siRNA. The observed p53 bands migrated at the expected molecular weight of approximately 53 kDa, as indicated by the molecular weight marker.

Densitometric analysis further validated the knockdown efficiency and demonstrated the antibody's ability to specifically detect p53 under our experimental conditions. Importantly, p53 expression was not decreased in cells treated with non-targeting siRNA, confirming the specificity of both the p53 siRNAs and the antibody detection. Interestingly, a slight increase in p53 levels was observed in the non-targeting siRNA group, which may reflect a mild cellular stress response potentially triggered by Lipofectamine transfection reagent, a known inducer of p53 expression in certain cell types. Collectively, these results confirm the reliability and specificity of the anti-p53 antibody used in our study.

2. the biotin-switch assay and its critical negative controls for specific detection of S-nitrosylated proteins

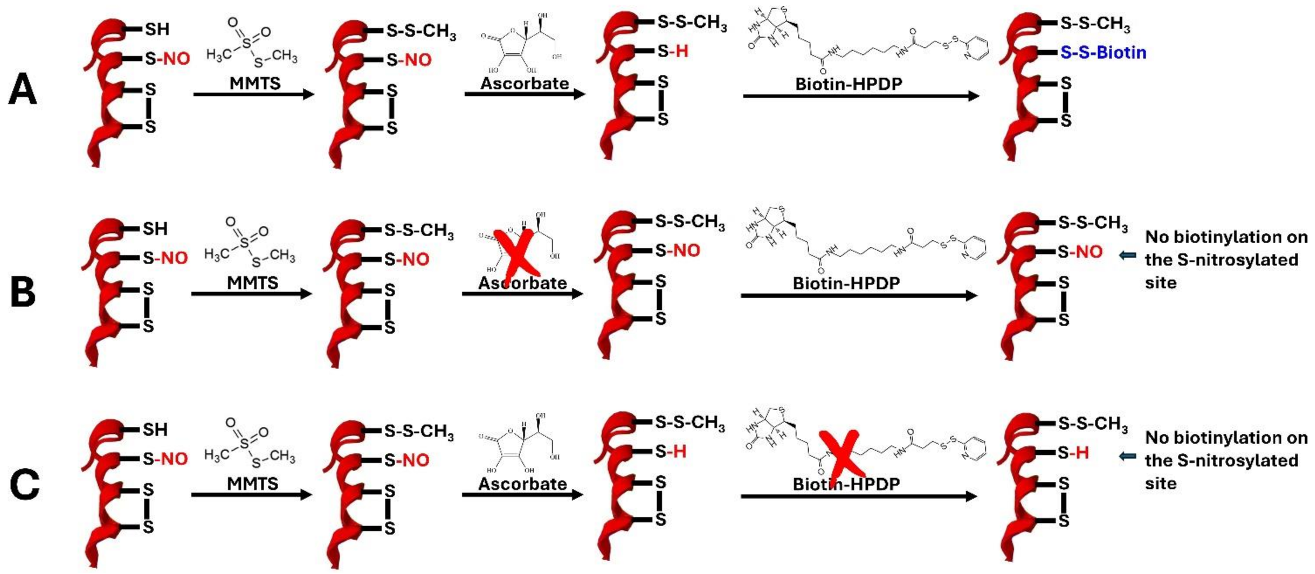

Supplementary Figure S1. Schematic representation of the biotin-switch assay and its critical negative controls for specific detection of S-nitrosylated proteins. (A) The complete biotin-switch assay workflow for specific detection of S-nitrosylated cysteine residues. First, all free thiol groups are irreversibly blocked using methyl methanethiosulfonate (MMTS). Then, ascorbate selectively reduces S-nitrosylated cysteines, regenerating free thiols specifically from S-nitrosylation (**SNO**). These thiols are subsequently labeled with the thiol-reactive biotinylating reagent biotin-HPDP, allowing for detection of SNO sites as biotin-tagged proteins. **(B)** Negative control omitting ascorbate. In the absence of ascorbate, S-nitrosylated cysteines are not reduced to free thiols and therefore cannot be labeled by biotin-HPDP. This control demonstrates that ascorbate reduction is essential for specific biotinylation of SNO sites and helps distinguish true SNO-derived signals from nonspecific biotin labeling. **(C)** Negative control omitting biotin-HPDP. Here, the assay includes MMTS blocking and ascorbate reduction, but lacks the biotinylating reagent. Although SNO groups are reduced to thiols, no biotinylation occurs. This control verifies that observed biotin signals are not due to endogenous biotinylated proteins or assay artifacts, further confirming the specificity and reliability of the assay. This comparative analysis

underscores the importance of appropriate negative controls in validating the specificity of the biotin-switch assay and avoiding misinterpretation of S-nitrosylation results.

3. Assessment of p53–DNA Binding under Nitrosative Stress via EMSA.

To evaluate the effect of nitrosative stress on p53 DNA-binding activity, we initiated a preliminary study to perform an Electrophoretic Mobility Shift Assay (EMSA) using nuclear extracts from A375 melanoma cells treated with or without 100 µM DETA NONOate for 24 hours. A radiolabeled, double-stranded DNA oligonucleotide containing the conserved human p53 consensus binding motif (5'-CGCAAACGATAGAACATGCCCGGGCATGTCCAGGG-3') was used as the probe, as previously reported [PMID: 1588974]. As a negative control, a 30-bp DNA fragment derived from the coding region of the β-actin gene (5'-CTCCGGCATGTGCAAGGCCGGCTTCGCGGG-3') was used to assess non-specific binding.

The DNA oligonucleotides were 5'-end labeled with [γ-^{32}P]ATP using T4 polynucleotide kinase, followed by heat inactivation and purification with Bio-Spin 6 columns (Bio-Rad). Double-stranded DNA was generated by annealing equimolar complementary strands in annealing buffer (10 mM Tris-HCl, pH 7.5, 10 mM NaCl) by heating at 75 °C for 15 minutes and slowly cooling to room temperature.

Nuclear extracts were incubated with the radiolabeled DNA in EMSA binding buffer (12 mM HEPES, 4 mM Tris-HCl, 1 mM EDTA, 5% glycerol, 1 mM DTT, 0.5 mg/mL BSA, and 1.5 mM $MgCl_2$) at 37 °C for 1 hour. Protein–DNA complexes were resolved on 5% non-denaturing polyacrylamide gels in 0.5× TBE buffer at 4 °C for 1 hour at 150 V, and the gels were then subjected to autoradiography.

As shown in **Supplementary Figure S3A**, nuclear extracts from untreated A375 cells formed a clear DNA–p53 complex with the consensus p53-binding probe, while DETA NONOate treatment reduced the formation of this complex. Western blot analysis confirmed comparable levels of total nuclear p53 in both groups. Quantification of DNA-binding activity relative to total nuclear p53 (**Supplementary Figure S3B**) revealed a significant reduction (*p ≤ 0.05) in DNA-binding efficiency following nitrosative stress. In contrast, no p53-DNA binding was observed using the nonspecific β-actin DNA fragment, regardless of treatment (**Supplementary Figure S3C**), confirming the sequence-specific nature of p53 binding.

These findings demonstrate that nitrosative stress impairs the DNA-binding activity of nuclear p53 in melanoma cells under nitrosative stress.

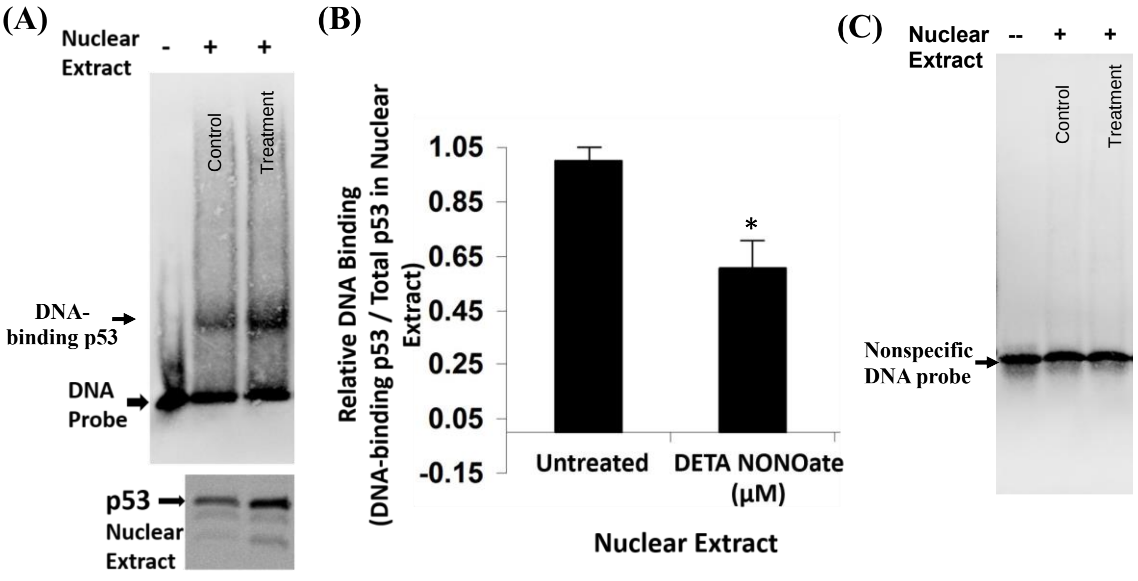

Supplementary Figure S3. Nitrosative stress impairs p53 DNA-binding activity in A375 melanoma cells. (A) EMSA showing nuclear extracts from untreated and 100 µM DETA NONOate-treated A375 cells incubated with a [γ-^{32}P]-labeled double-stranded oligonucleotide containing the consensus p53-binding sequence. The top panel shows DNA–p53 complex formation; the bottom panel displays total p53 protein levels in nuclear extracts as assessed by Western blot. (B) Quantification of

DNA-bound p53 normalized to total nuclear p53, expressed as mean ± SEM from at least three independent experiments. *p ≤ 0.05 compared to untreated control. (C) EMSA using the same nuclear extracts incubated with a radiolabeled nonspecific DNA probe (30-bp fragment from the β-actin coding sequence). No specific p53 binding was observed with the nonspecific probe, confirming binding specificity.

4. Nitrosative Stress Differentially Modulates p53 Downstream Target Expression in Melanoma Cells

Given that p53 undergoes S-nitrosylation in response to nitrosative stress, we conducted a preliminary investigation to evaluate how this redox modification influences the expression of key p53-regulated downstream targets. Specifically, we focused on the pro-apoptotic protein PUMA, the cyclin-dependent kinase inhibitor p21, and the E3 ubiquitin ligase MDM2, well-established transcriptional targets of p53 [PMID: 16991100, 23150757, 27156098]. A375 and SB2 melanoma cell lines were treated with increasing concentrations (20, 50, and 100 µM) of two nitric oxide (NO) donors, DETA NONOate and S-nitrosoglutathione (GSNO), for 24 hours. Following treatment, total protein was extracted and analyzed by Western blotting. Expression levels of p53 and its downstream effectors were quantified by densitometry and normalized to β-actin as a loading control.

As shown in **Supplementary Figure S4**, both NO donors significantly upregulated p53, p21, and MDM2 expression in a dose-dependent manner in both A375 and SB2 cells (p ≤ 0.05). Interestingly, PUMA expression exhibited a cell line-dependent response: while no significant changes were observed in A375 cells, SB2 cells showed a marked reduction in PUMA protein levels at 50 µM and 100 µM concentrations of both GSNO and DETA NONOate (p ≤ 0.05). These findings suggest that nitrosative stress modulates p53 transcriptional activity in a gene- and context-specific manner, potentially altering cell fate decisions in melanoma.

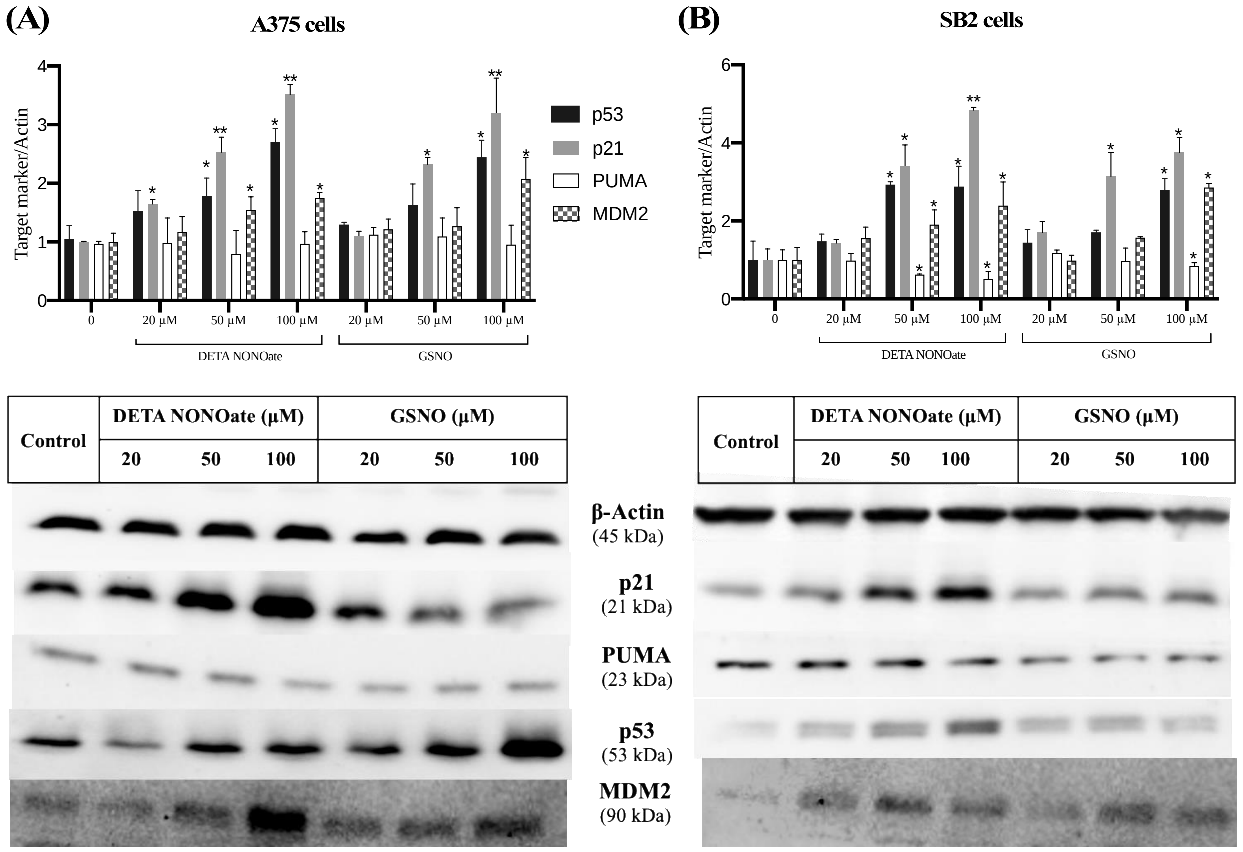

Supplementary Figure S4. Effect of nitric oxide (NO) donors GSNO and DETA NONOate on p53 downstream targets in melanoma cells. (A) A375 and (B) SB2 cells were treated with increasing concentrations (20, 50, and 100 µM) of DETA NONOate or GSNO for 24 hours. Western blotting was performed to detect p53, p21, PUMA, and MDM2 protein levels, with β-actin used as a loading control. The bar graphs represent densitometric quantification of protein expression normalized to β-actin, expressed relative to untreated controls (set to 1). Representative blots are shown below each graph. Data represent mean ± SEM from at least three independent experiments. p ≤ 0.05, p ≤ 0.01 versus untreated control.

5. Preliminary Screening of Melanoma Tumors with High Nitrosative Stress for Future S-Nitrosylation Studies.

Due to the inherent instability and short half-life of SNO modifications in biological systems, detecting endogenous S-nitrosylated proteins in tumor tissues, especially in archived or formalin-fixed paraffin-embedded (FFPE) samples, remains technically challenging. To circumvent this limitation and prioritize suitable tumor specimens for future SNO-proteomic analysis, we employed immunohistochemistry (IHC) using an anti-nitrotyrosine antibody (Abcam, Cat. #ab125106) as a surrogate marker of nitrosative stress. Protein tyrosine nitration, unlike SNO, is a more stable and reliably detectable post-translational modification that correlates with elevated nitric oxide (NO) and peroxynitrite levels in the tumor microenvironment [PMID: 23157446].

As illustrated in **Supplementary Figure S5**, we were able to clearly differentiate between melanoma tumors exhibiting high versus low levels of nitrotyrosine staining. Tumors with elevated nitrotyrosine signals are presumed to have experienced higher nitrosative stress, making them strong candidates for harboring higher levels of S-nitrosylated proteins, including SNO-p53. These high-nitrotyrosine tumors will serve as priority samples for future biotin-switch assays and mass spectrometry-based detection of SNO modifications, provided they are collected fresh or snap-frozen to preserve labile redox modifications. We are currently assembling a cohort of 20 high-nitrotyrosine and 20 low-nitrotyrosine melanoma specimens, with a focus on obtaining freshly frozen tumor tissues in sufficient quantities to enable rigorous SNO-proteomic analyses.

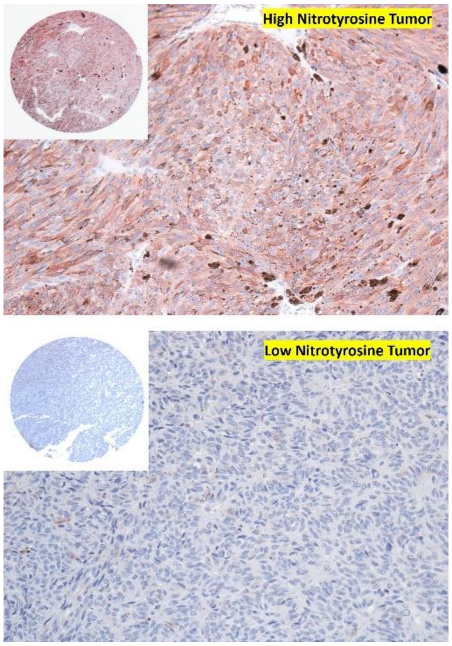

Supplementary Figure S5. Representative immunohistochemical staining of melanoma tumor tissues for nitrotyrosine to assess nitrosative stress levels.

Top: Tumor section classified as high nitrotyrosine shows widespread and intense cytoplasmic and perinuclear staining, indicative of elevated nitrosative stress. Bottom: Tumor section classified as low nitrotyrosine demonstrates minimal staining, consistent with reduced nitrosative stress.

Tissue sections were stained using an anti-nitrotyrosine antibody (Abcam, Cat. #ab125106), and counterstained with hematoxylin. These results serve as a basis for selecting tumors suitable for downstream S-nitrosylation analyses such as the biotin-switch assay and LC-MS/MS-based proteomics. Insets show low-magnification views of the corresponding tissue microarrays.
